# Supplementary material for: Polyketide synthase gene clusters in the ericoid mycorrhizal fungus Oidiodendron maius: genome-wide prediction, expression profiling, and functional characterization of knockout mutants
Source: Front Plant Sci. 2026 Sep 16;17:1924430. doi: 10.3389/fpls.2026.1924430 (PMC13623903; doi:10.3389/fpls.2026.1924430)
Supplement: Supplementary file 1 [file SupplementaryFile1.zip › Supplementary Materials/Supplementary Folder 1.zip!/Oidma1_AssemblyScaffolds_Repeatmasked/knownclusterblast/cluster5/jgi.p_Oidma1_22802_mibig_hits.html]

| MIBiG Protein | Description | MIBiG Cluster | MiBiG Product | % ID | % Coverage | BLAST Score | E-value |
| --- | --- | --- | --- | --- | --- | --- | --- |
| EWG54274.1 | hypothetical\_protein | BGC0001190 | Fusaric\_acid\_biosynthetic\_gene\_cluster | 31 | 102.228412256 | 494 | 2.7e-139 |
| AAS98174.1 | ochratoxin\_A\_non-ribosomal\_peptide\_synthetase | BGC0001030 | Ochratoxin\_A\_biosynthetic\_gene\_cluster | 34 | 63.7883008357 | 374 | 3.1e-103 |
| BAV19380.1 | NRPS-like\_enzyme | BGC0001390 | LL-Z1272beta\_biosynthetic\_gene\_cluster | 26 | 97.4930362117 | 257 | 9.3e-68 |
| EAA65602.1 | hypothetical\_protein | BGC0000022 | Asperfuranone\_biosynthetic\_gene\_cluster | 29 | 40.2042711235 | 144 | 8.8e-34 |
| EHA28237.1 | hypothetical\_protein | BGC0001143 | Azanigerone\_biosynthetic\_gene\_cluster | 30 | 44.1968430826 | 142 | 3.4e-33 |
| ALI92655.1 | CitS\_citrinin\_polyketide\_synthase | BGC0001338 | Citrinin\_biosynthetic\_gene\_cluster | 29 | 37.1402042711 | 137 | 1.1e-31 |
| AGN71604.1 | conidial\_yellow\_pigment\_biosynthesis\_polyketide\_synthase | BGC0000027 | Azaphilone\_biosynthetic\_gene\_cluster | 29 | 36.9545032498 | 134 | 7e-31 |
| ADH01663.1 | putative\_polyketide\_synthase\_PKS3 | BGC0000099 | Monascorubrin\_biosynthetic\_gene\_cluster | 28 | 35.747446611 | 130 | 1.3e-29 |
| CAP95404.1 |  | BGC0001404 | Sorbicillin\_biosynthetic\_gene\_cluster | 29 | 32.6833797586 | 110 | 1.4e-23 |
| ACS68554.1 | hybrid\_PKS-NRPS\_protein | BGC0001026 | NG-391\_biosynthetic\_gene\_cluster | 25 | 23.3983286908 | 63 | 1.5e-09 |
